# Supplementary material for: User-Centered Redesign of Monitoring Alarms: A Pre–Post Study on Perception, Functionality, and Recognizability Following Real-Life Clinical Implementation
Source: Healthcare (Basel). 2025 Nov 24;13(23):3033. doi: 10.3390/healthcare13233033 (PMC12692177; doi:10.3390/healthcare13233033)
Supplement: Supplementary file 1 [file healthcare-13-03033-s001.zip › Multimedia Supplement 3 Psychometric Analysis of Likert Scales and Sensitivity analysis restricted to participants with no training.pdf]

Multimedia Supplement 3

1. **Psychometric Analysis of Likert Scales**

To evaluate the internal consistency and dimensionality of the scales, we conducted the following analyses using JASP version 0.95.4 (Apple Silicon):

1. **Internal consistency:** Coefficient ω was calculated for both scales. The Perceived Functionality scale (6 items) showed moderate reliability (ω = 0.652), while the Perceived Sound Appeal scale (5 items) showed lower reliability (ω = 0.489).
2. **Exploratory factor analysis (EFA):** A one-factor EFA was conducted for each scale to examine the underlying factor structure. Factor loadings and uniqueness values were extracted to assess how well each item represents the underlying construct.
3. **Interpretation:** The Functionality scale largely loaded onto a single factor, indicating a coherent construct. The Sound Appeal scale displayed lower internal consistency and some items with high uniqueness, suggesting it captures broader or more heterogeneous aspects of sound appeal, potentially reflecting differences in individual preferences or understanding.

These results are provided to support transparency and to inform future work, which may consider item response theory or ordinal modeling for further validation.

Table S1: Unidimensional Reliability Perceived Sound Appeal

Frequentist Scale Reliability Statistics

Coefficient	Estimate	Std. Error	95% CI	
			Lower	Upper
Coefficient ω	0.489	0.058	0.375	0.603

Note. The following item correlated negatively with the scale: Sensibility5.

Frequentist Individual Item Reliability Statistics

Item	Coefficient ω (if item dropped)		
	Estimate	Lower 95% CI	Upper 95% CI
Sensibility1	0.400	0.266	0.534
Sensibility2	0.260	0.072	0.447
Sensibility3	0.334	0.182	0.485
Sensibility4	0.523	0.412	0.635
Sensibility5	0.622	0.531	0.714

Tabel S2: Exploratory Factor Analysisn Perceived Sound Appeal

Chi-Squared Test

	Value	df	p
Model	7.766	5	.170

Factor Loadings

	Factor 1	Uniqueness
Sensibility2	0.845	0.286
Sensibility3	0.709	0.498
Sensibility1	0.537	0.712
Sensibility4		0.959
Sensibility5		0.880

Note. Applied rotation method is promax.

Table S3: Unidimensional Reliability Perceived Functionality

Frequentist Scale Reliability Statistics

			95% CI	
Coefficient	Estimate	Std. Error	Lower	Upper
Coefficient ω	0.652	0.041	0.571	0.733

Note. The following item correlated negatively with the scale: Functionality 1.

Frequentist Individual Item Reliability Statistics

	Coefficient ω (if item dropped)		
Item	Estimate	Lower 95% CI	Upper 95% CI
Functionality2	0.500	0.387	0.613
Functionality3	0.509	0.397	0.620
Functionality4	0.616	0.527	0.706
Functionality5	0.610	0.519	0.701
Functionality6	0.536	0.431	0.641

Frequentist Individual Item Reliability Statistics

Item	Coefficient ω (if item dropped)		
	Estimate	Lower 95% CI	Upper 95% CI
Functionality 1	0.787	0.734	0.840

Table S4: Exploratory Factor Analysis Perceived Functionality

Chi-Squared Test

	Value	df	p
Model	37.082	9	< .001

Factor Loadings

	Factor 1	Uniqueness
Functionality2	0.843	0.290
Functionality3	0.842	0.290
Functionality6	0.801	0.359

Factor Loadings

	Factor 1	Uniqueness
Functionality 1	0.748	0.440
Functionality5	0.550	0.698
Functionality4	0.539	0.710

Note. Applied rotation method is promax.

1. Sensitivity analysis restricted to participants who did not attend training lecture

To address potential bias from prior exposure to the refined alarm sounds, a sensitivity re-analysis was conducted excluding participants who reported attending the January 2024 training session. Two participants with inconsistent responses regarding training attendance between the pre- and post-phase were also excluded. In total, 41 of the original 77 participants were included in this restricted sample. The mixed Poisson and linear mixed regression models for *Perceived Sound Appeal*, *Perceived Functionality*, and *Recognizability* were re-estimated using this subsample. Across all three outcomes, the results closely matched those of the full-sample analysis, indicating that exclusion of trained participants did not materially affect the findings.

For *Perceived Sound Appeal*, the post-phase coefficient was 0.58 points (95% CI: 0.37–0.80; $P < 0.001$), compared to 0.51 points (95% CI: 0.37–0.64; $P < 0.001$) in the main analysis.

For *Perceived Functionality*, the post-phase coefficient was –0.21 (95% CI: –0.39 to –0.02), compared to –0.15 (95% CI: –0.27 to –0.03).

For *Recognizability*, the rate ratio was 0.93 (95% CI: 0.79–1.09), compared to 0.93 (95% CI: 0.83–1.04) in the main analysis.
