# Supplementary material for: User-Centered Redesign of Monitoring Alarms: A Pre–Post Study on Perception, Functionality, and Recognizability Following Real-Life Clinical Implementation
Source: Healthcare (Basel). 2025 Nov 24;13(23):3033. doi: 10.3390/healthcare13233033 (PMC12692177; doi:10.3390/healthcare13233033)
Supplement: Supplementary file 1 [file healthcare-13-03033-s001.zip › Multimedia Supplement 1 Audio samples of traditional and refined Philips alarm sounds used in the pre- and post-intervention phases of the study.pptx]

## Slide 1
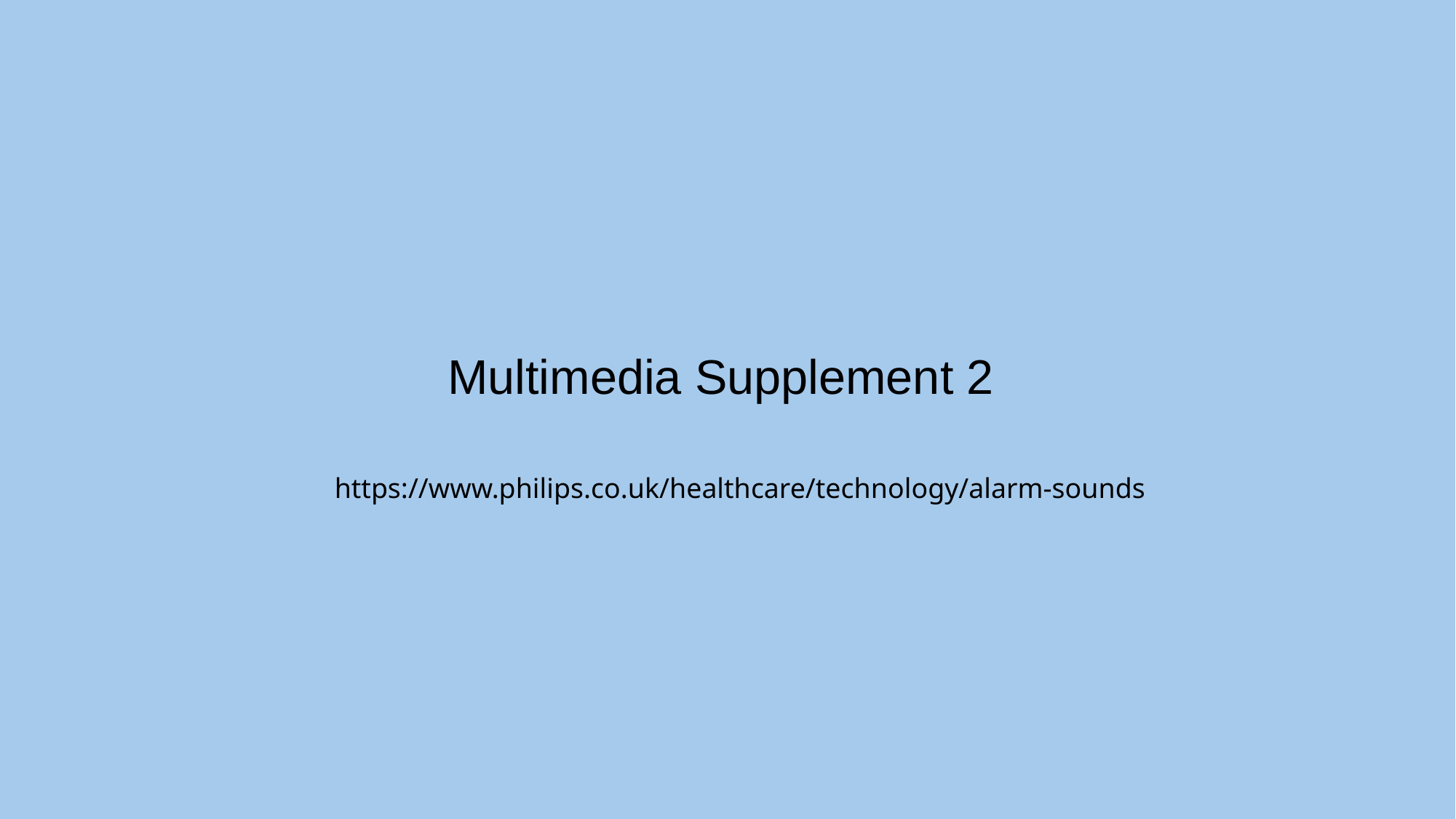

# Multimedia Supplement 2
https://www.philips.co.uk/healthcare/technology/alarm-sounds

## Slide 2
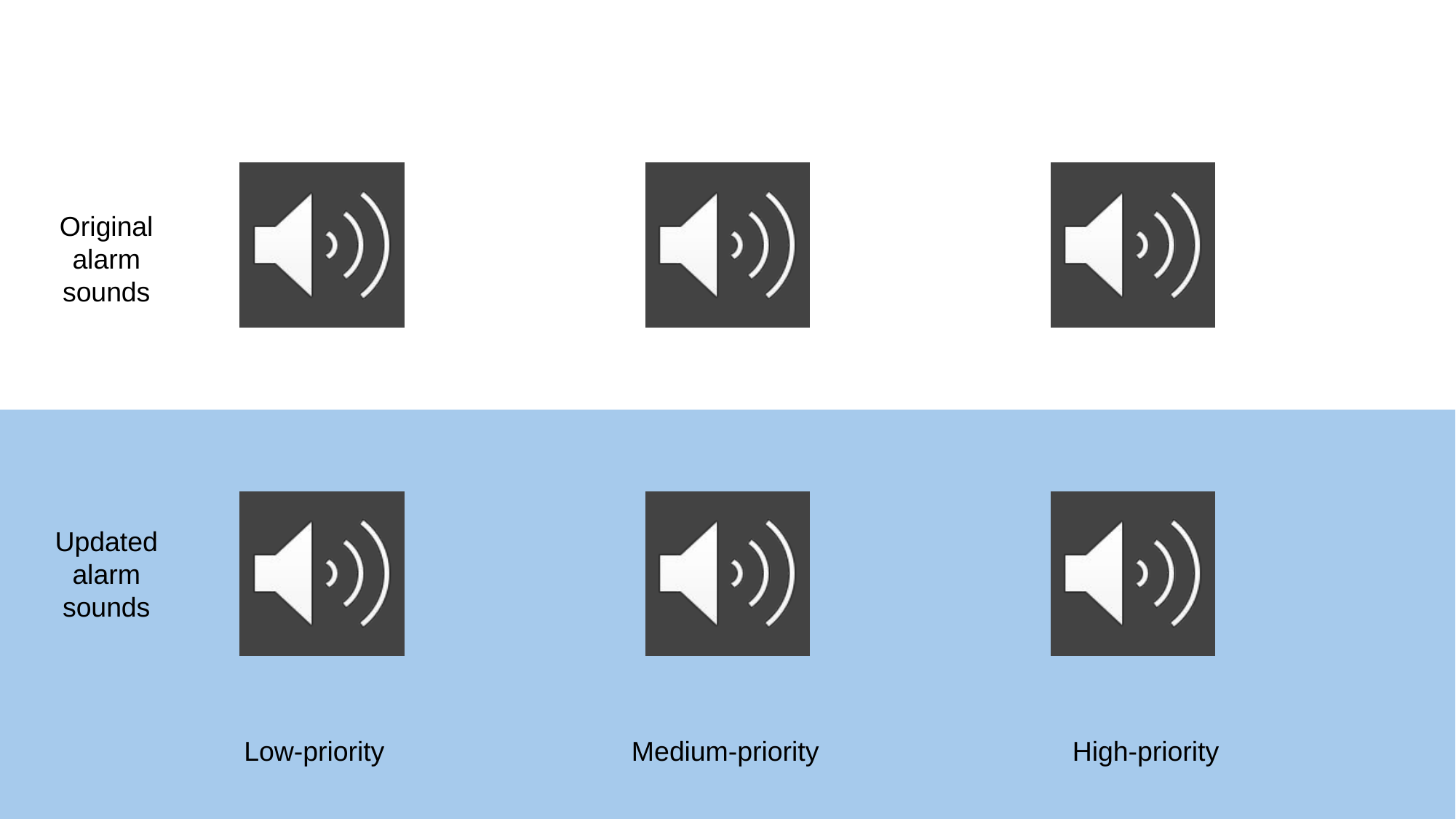

Original alarm sounds
Updated alarm sounds
Low-priority
Medium-priority
High-priority
