# Supplementary material for: User-Centered Redesign of Monitoring Alarms: A Pre–Post Study on Perception, Functionality, and Recognizability Following Real-Life Clinical Implementation
Source: Healthcare (Basel). 2025 Nov 24;13(23):3033. doi: 10.3390/healthcare13233033 (PMC12692177; doi:10.3390/healthcare13233033)
Supplement: Supplementary file 1 [file healthcare-13-03033-s001.zip › Multimedia Supplement 2 Description of the design process for the refined Philips alarm sounds.pdf]

Description of Design Process

To clarify from the outset: our work is a clinical before-and-after intervention study, conducted by anesthesiologists at the University Hospital of Zurich. Our role was to evaluate the Refined Alarm Sounds in daily clinical practice. We were not involved in the creation or design of the alarm sounds themselves, which were independently developed by Philips and Sen Sounds. Consequently, the design process was not the main focus of our study. Nevertheless, in consultation with Sen Sounds, we provide an overview of the design process to the best of our knowledge, in order to give readers additional context.

Timeline and Process

Research and design occurred in 2 rounds over 6 months. Each design round followed a diverging and converging pattern: qualitative methods such as interviews and workshops informed the initial exploration, while quantitative surveys evaluated refined options. The process spanned 39 semi-structured interviews and five virtual workshops across 12 countries, engaging over 100 participants, primarily nurses and anesthesiologists, with additional input from ICU physicians, patients, and other stakeholders. Feedback cycles spanned open-ended discussions and closed-ended survey responses, enabling iterative refinement of tone designs. Criteria for “better” alarms were derived from the interviews and workshops, then these criteria served as indicators by which alternative tones were compared and assessed in surveys (via pairwise comparisons).

Adjustments and Goals

Key audio parameters—pitch, timbre, frequency, amplitude envelope, and interval between tones—were systematically adjusted to address user feedback. Participants expressed preferences for tones that were “softer,” “less shrill,” and “more natural,” especially for commonly heard low-priority alarms. Goals for the redesigned tones (i.e. what “better” means) included reducing auditory fatigue, improving audibility and distinguishability, and minimizing distress for both clinicians and vulnerable patient populations, such as infants or individuals with PTSD.

Specific changes included:

- **Low-Priority Tones:** Enhanced with a "rounder" amplitude envelope featuring non-instantaneous attacks and longer decays, resulting in more percussive and natural tones. Interval between tones extended.
- **Medium Priority Tones:** Pitch increased to make it different from (and thus more distinguishable from) low-priority tones, but still lower pitch than high-priority tones, given that pitch can be perceived as an indicator of urgency.
- **High-Priority Tones:** Modified to reduce the perceptual loudness caused by amplified harmonics, addressing concerns about harshness and creating a more balanced timbre without sacrificing urgency.

These modifications also accounted for hardware constraints (e.g., the IntelliVue speaker's frequency response) and human auditory perception.

Evaluation Steps

Data collection and analysis were integral to tone refinement. Qualitative insights from interviews and workshops were coded and synthesized into typological models, highlighting key design factors and preferences. Quantitative evaluation used surveys to compare tone options, isolating individual parameters to assess their impact on participant preferences. Simple counts were used to understand preferences between options on each criterion (see Figures 6 and 7).

- **First Survey (n=98):** Presented a wide variety of multiple tone options, each embedded in hospital ambient recordings, for comparative evaluation across criteria like audibility, distinguishability, annoyance and fatigue. Participant feedback informed the elimination of certain options and the refinement of others. 26 tone options (including Philips original tones and IEC recommended tones) were provided across 18 pairwise comparison questions; each comparison isolated a difference in a specific parameter (e.g. pitch or amplitude envelope).
- **Second Survey (n=25):** Compared the final proposed tones against the original tones, focusing on user preferences for overall improvement. Each priority level was presented as a pairwise comparison between proposed and original tones across 4 questions: one for each priority, then all priorities together.

Feedback interpretation relied on participant-provided descriptions, analogies, and mimicry to translate subjective impressions into actionable design changes.

Outcomes

This participatory process facilitated the creation of alarm tones better aligned with user needs, reducing the psychological burden on clinicians and enhancing patient experiences. By embedding sound design within a structured research framework, the study advanced a more empathetic and effective approach to medical device innovation.

Figures and Tables

How do we make tones better and what does "better" mean?

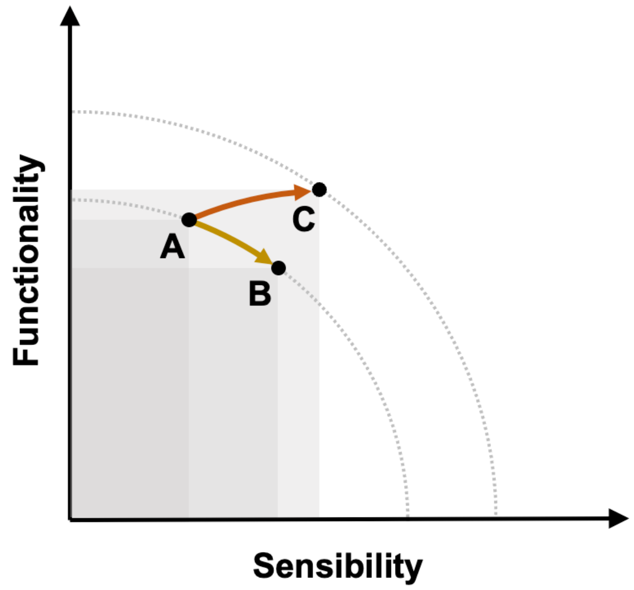

Functionality

- More easily heard and attention-getting (stands out above the background)
- More distinct from other sounds (not mistaken for something else)
- Sounds more like what it means (low, medium, or high priority)
- Better stimulates a prompt response (not ignored or unattended)
- Easier to isolate (among possible locations if could be coming from)

Sensibility

- Less startling or aggravating (beyond what is necessary to get attention)
- Less fatiguing to hear over time (everyday, possibly for many years)
- Less distressing for patients to hear (either short or long term)
- Not distracting from important conversations (with clinicians or family)
- More pleasant to me (given my personal taste)

Figure S1: What does it mean to sound "better?" Conventional wisdom assumes that, in order to make a tone more sensible, its functionality must be sacrificed. This is illustrated here by a shift from point A to B along a production possibilities curve (Bloomenthal 2021 [1])*, representing a constrained trade off. However, our research suggests that the trade off need not be so constrained. Through careful design and training, it may be possible to move from a more constrained to a less constrained trade off space, enabling a shift from point A to point C.*

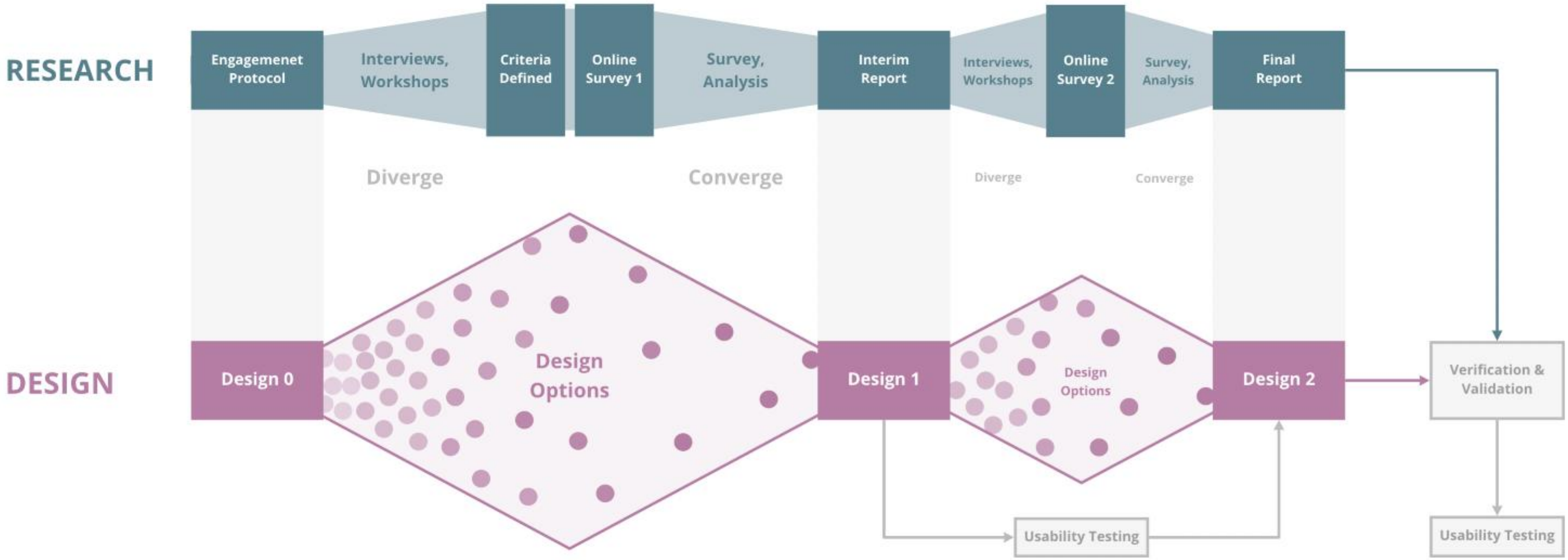

Figure S2: After conducting a literature review, a speaker hardware analysis, and establishing general constraints, the research and design process took a "double diamond" approach. Each diamond represents a design iteration. In the "divergent thinking" phase of each iteration, open-ended input was gathered and synthesized with participants though interviews with individuals and workshops with groups, and sound design concurrently responded to this input by exploring a growing number of alarm tone options. In the "convergent thinking" phase of each iteration, alarm tone options were narrowed down to include a limited number for inclusion in surveys, participants answered close-ended questions (largely pairwise comparisons), and survey data were analysed to inform the final prototype tone set for that iteration. "Design 0" was the initial prototype tone set that was used alongside the original tones for listen-and-react questions in the first set of engagements, "Design 1" was used this way in the second set of engagements, and "Design 2" was the final set that went into verification and validation processes at Philips.

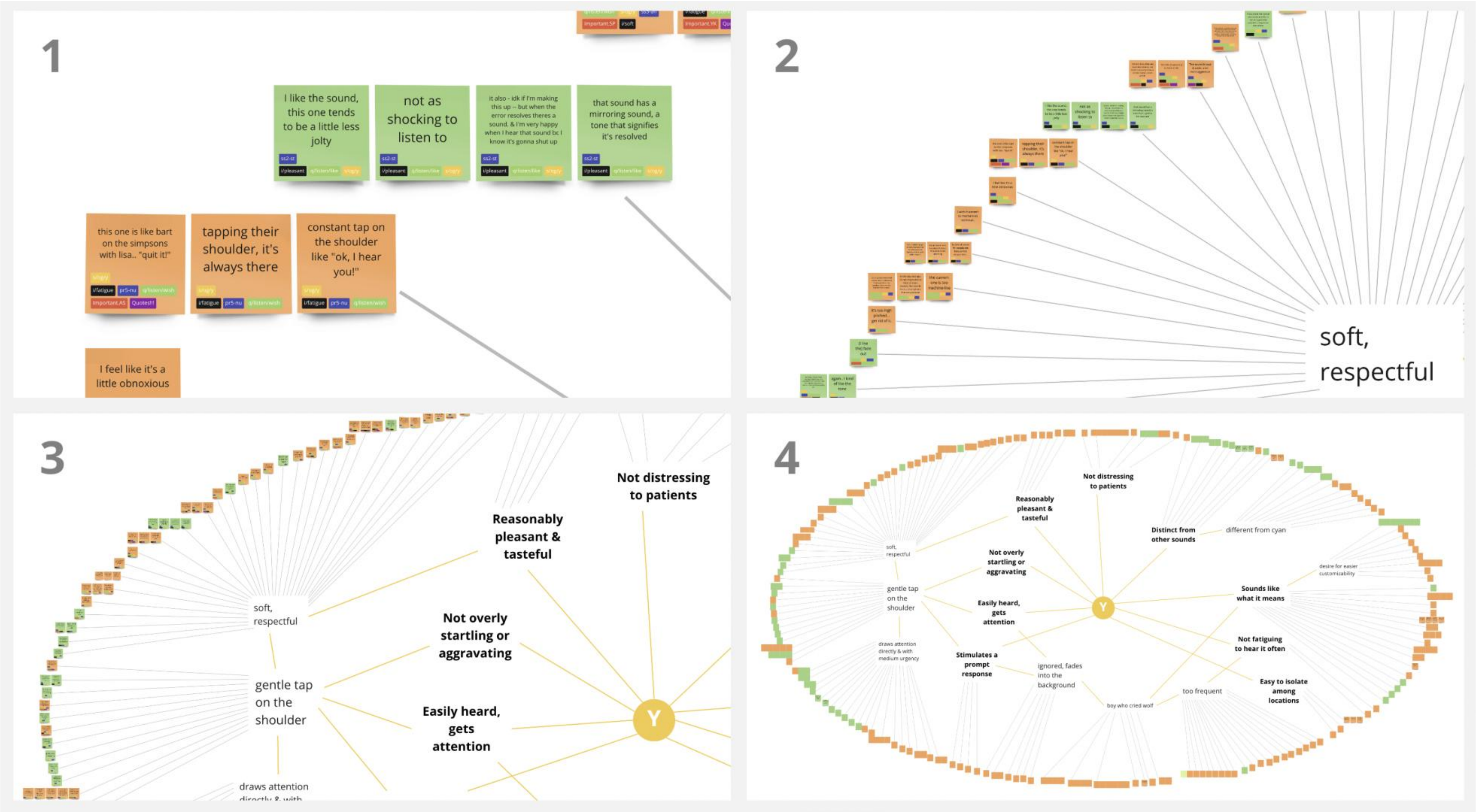

Figure S3: Example of coded excerpts and clustering for comments about what is important regarding the medium priority (yellow) alarm tone, zooming out from position 1 (individual excerpts) to position 4 (full typological model).

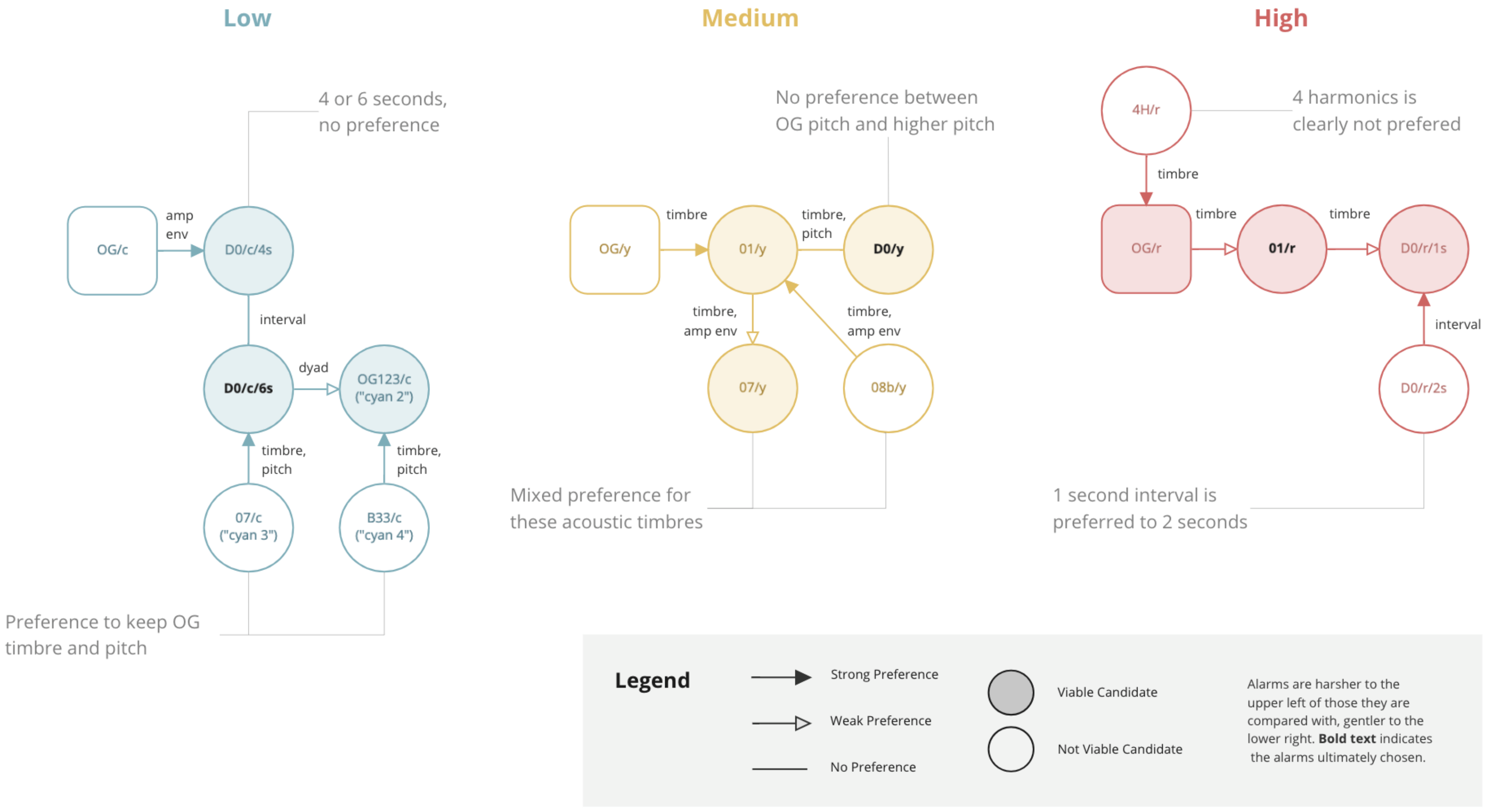

Figure S4: Comparisons and relative preferences among alarm tone options, per "All Things Considered" question in survey 1. Each question compared a specific difference on one or two parameters, such as pitch and timbre. Sound names start with their timbral "family" and include other parameter information as well. "OG" is the original sound family, while "D0," "01," "07," "08," "B33," "OG123," and "4H" are prototype alarm families created for this project. For each family, "/c" is a cyan (low priority) alarm tone, "/y" is a yellow (medium priority) alarm tone, and "/r" is the red (high priority) alarm tone. "/1s," "/2s," "/4s," and "/6s" represent the interval length for each tone.

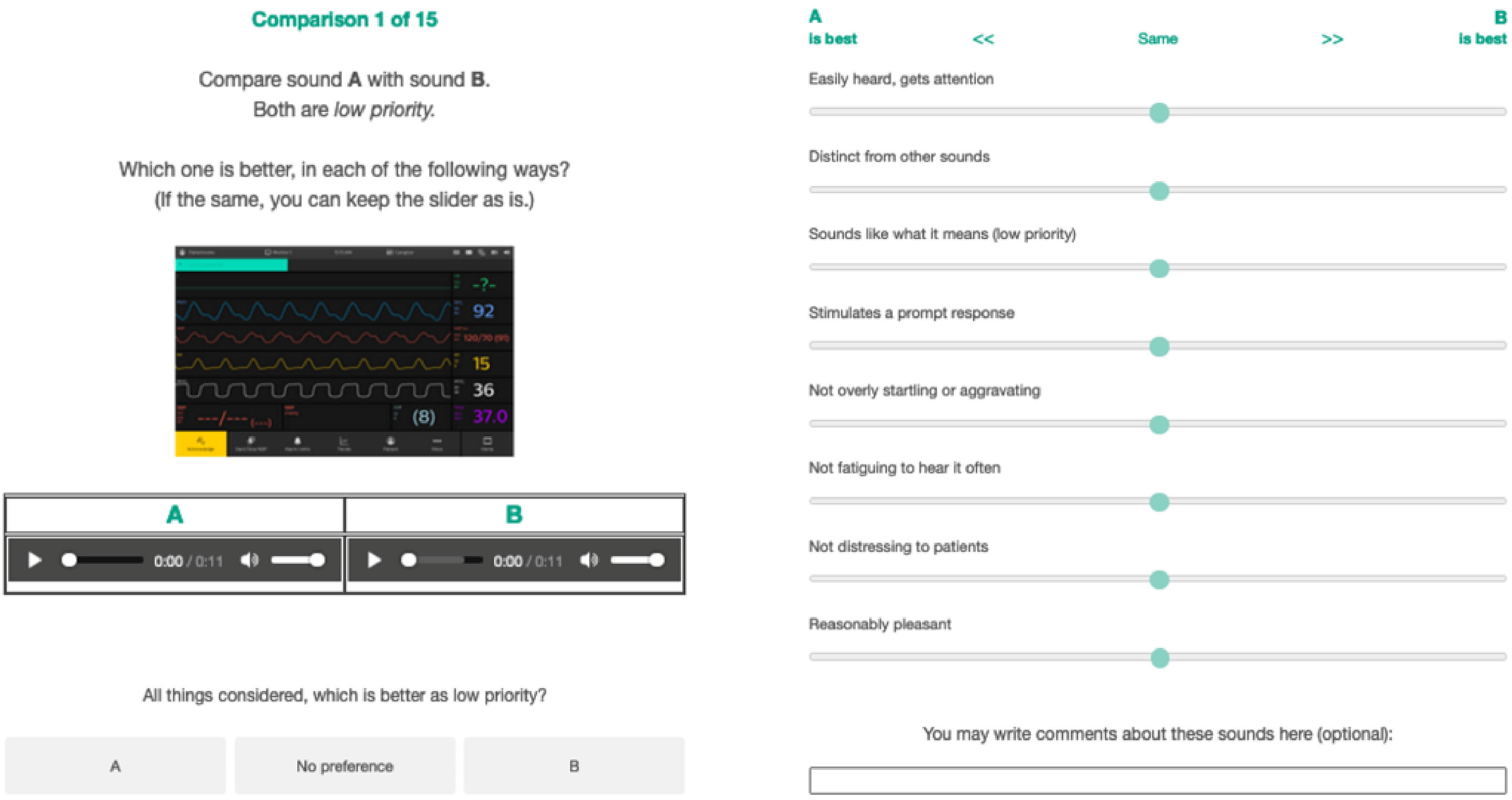

Figure S5: Example of question language and format for both surveys. Audio files were embedded in the survey page, with sound "A" on the left and "B" in the right. Actual sound designations were not displayed for the survey taker. Participants could move sliders in either direction to state preferences independently for each of 8 criteria. The position was captured on a 10 point scale in either direction (-10 to +10). Participants also were asked to provide an overall preference, A or B, and to write any comments they might have.

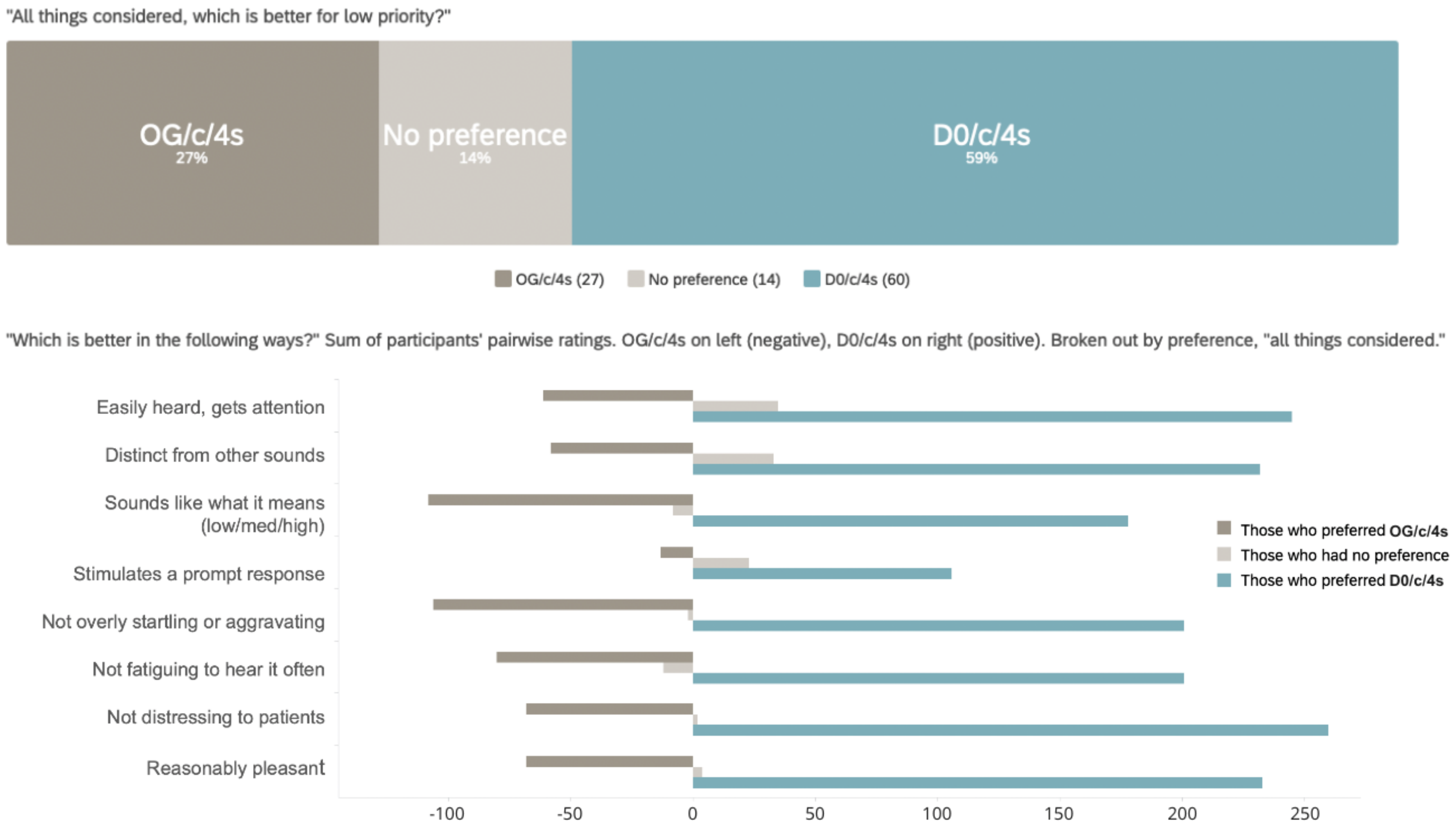

Figure S6: Participant preferences for two low priority alarms differing only in amplitude envelope, original ("OG") versus new ("D0"). For each criterion, preferences for the original extend to the left, and preferences for the new extend to the right.

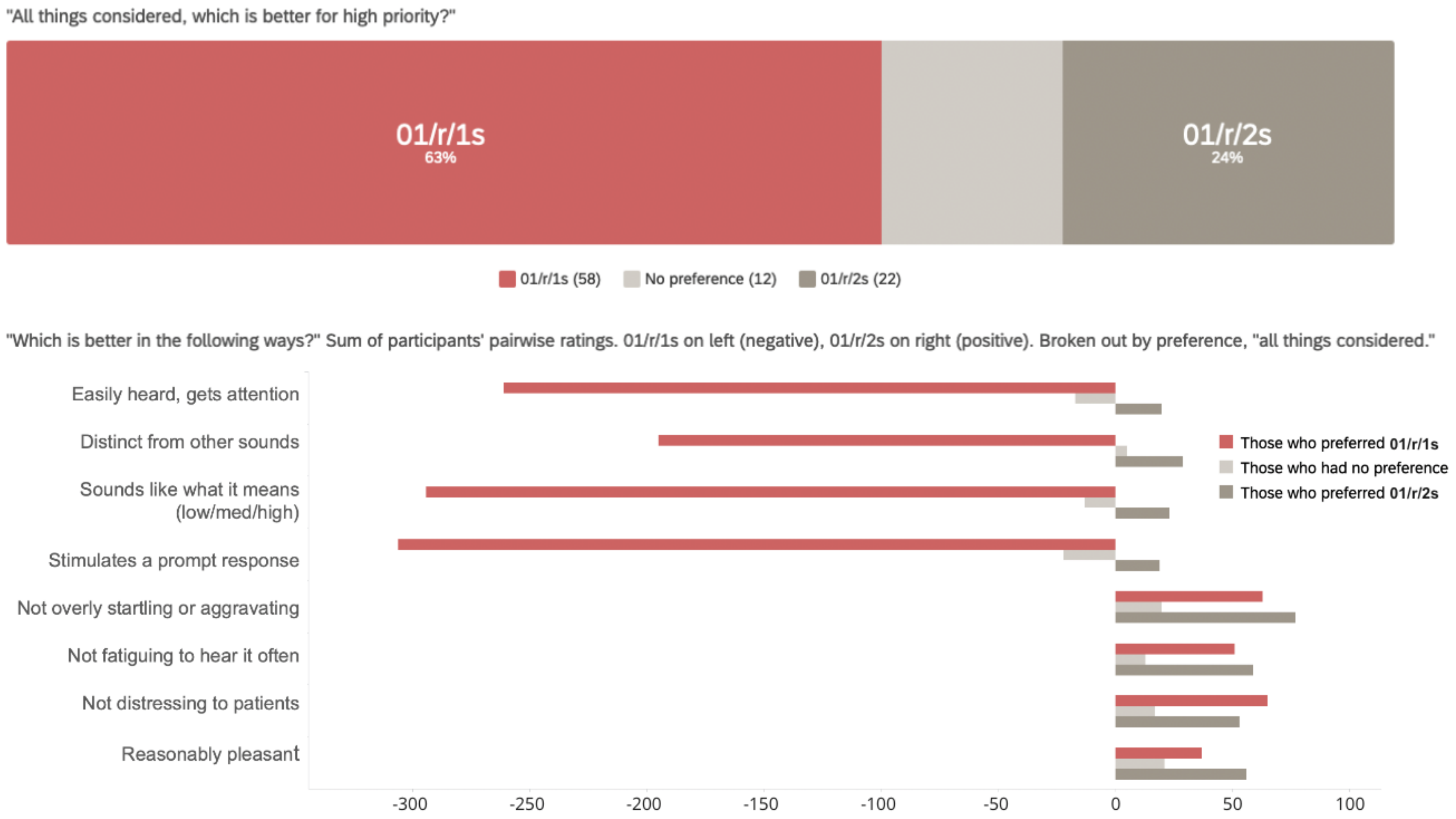

Figure S7: Participant preferences for two high priority alarms differing only in interval, 1 second versus 2 seconds. For each criterion, preferences for the 1 second version extend to the left, and preferences for the 2 second version extend to the right

Table S1: Alarm priorities and their meanings. (Definitions per International Electrotechnical Commission, 2020, pp.13-14)

Priority	Color	Definition	Example condition
**Low**	Cyan	Operator awareness is required and future action might be needed. Awareness implies the planning of future workflow is expected.	• Failure of an infusion pump for maintenance of intravenous fluids<br>• Failure of an enteral feeding pump<br>• Failure of a patient weighing system
**Medium**	Yellow	Prompt operator response is required. Prompt implies the re-planning of current workflow is expected.	• High or low blood pressure<br>• Mild hypoxemia<br>• High or low pCO2
**High**	Red	Immediate operator response is required. Immediate implies the interruption of current workflow is expected.	• Asystole<br>• Ventricular fibrillation<br>• Extreme hypoxemia

Table S2: Participants demographics in each engagement. All interviews and workshops were conducted virtually over Zoom. Dashes indicate no data available. (Note: numbers for workshops represent those who contributed input via online webform during the event, and which was formally synthesized. However, there were more who attended and whose voices were heard.)

	Interviews	Workshops	Survey 1	Survey 2
**Responsibility**				
I am/was directly responsible for attending to patient monitoring alarms	32	---	91	23
I have never been directly responsible for attending to patient monitoring alarms.	7	---	7	2
**Experience with patient monitoring alarms**				
Nurse	21	25	60	23
Physician	4	3	8	0
Anesthesiologist	4	1	15	0
Medical student or resident	1	0	1	1
Another healthcare worker (paramedic, chaplain, secretary)	3	5	7	1
Not a healthcare worker (patient, family caregiver, device engineer, device marketer)	6	6	7	0
**Tenure in a clinical setting**				
0-5 years	6	2	13	3
6-10 years	5	9	20	4
11-15 years	7	8	18	8
16-20 years	0	2	10	0
20+ years	11	11	32	9
unknown (but more than 0 years)	4	1	0	0
never	6	7	5	1
**Location**				
North America (Canada, United States)	32	24	---	---
Europe (Germany, Netherlands, Switzerland, United Kingdom)	6	12	---	---
Asia (Japan)	1	4	---	---
**Total**	**39**	**40**	**98**	**25**

Table S3: Codes used for developing criteria that guided sound design. Sources for codes derived from literature are provided, otherwise labelled as emergent.

Code	Meaning	Source	Criterion in Final Phrasing
i/hear	I can hear it, it stands out above the rest	ANSI/AAMI, 2018 [2]; IEC, 2020 [3], C. L. Bennett et al., 2015 [4]; Hasanain et al., 2017 [5]; Bolton et al., 2019 [6]	More easily heard and attention-getting (stands out above the background)
i/attention	It gets my attention (or not), can't ignore (or can)	ANSI/AAMI, 2018 [2]; IEC, 2020 [3]	
i/means	I know what it means (or not)	ANSI/AAMI, 2018 [2]; IEC, 2020 [3]; Phansalkar et al., 2010 [7]; Gillard & Schutz, 2016 [8]; McDougall & Edworthy, 2018 [9]; McDougall et al., 2020 [10]	Sounds more like what it means (low, medium, or high priority)
i/confuse	It may (or may not) be confused with another sound	ANSI/AAMI, 2018 [2]; IEC, 2020 [3]; J. R. Edworthy et al., 2018 [11] McNeer et al., 2018 [12]; C. Bennett et al., 2019 [13]	More distinct from other sounds (not mistaken for something else)
i/action	It makes me move, take action (or not)	Emergent	Better stimulates a prompt response (not ignored or unattended)
i/find	I can find it, isolate it among others (or not)	ANSI/AAMI, 2018 [2]; IEC, 2020 [3]; J. Edworthy et al., 2018 [11]	Easier to isolate (among possible locations if could be coming from)
i/soft	It is soft, gentle, round (or not), aggressive (or not)	Emergent	Less startling or aggravating (beyond what is necessary to get attention)
i/startle	It is startling in the moment (or not)	ANSI/AAMI, 2018 [2]	
i/fatigue	It is stressing or fatiguing over time (or not)	IEC, 2020 [3]; Topf & Dillon, 1988 [14]; Topf, 2000 [15]; Morrison et al., 2003 [16]; Ryherd et al., 2008 [17]; J. P. Keller et al., 2011 [18]; Watson et al., 2015 [19]; Cho et al., 2016 [20]	Less fatiguing to hear over time (everyday, possibly for many years)
i/patient	It is startling or fatiguing to the patient (or not)	Topf, 2000 [15]; Basner, 2011 [21]; Shivers et al., 2013 [22]; Basner et al., 2014 [23]; Sakallaris et al., 2015 [24]; Sen & Sen, 2020 [25]	Less distressing for patients to hear (either short or long term)
i/distract	It is distracting (or not), I can (or can't) hear what I need	Murthy et al., 1995 [26]; Hasfeldt et al., 2010 [27]; S. Keller et al., 2016 [28]	Not distracting from important conversations (with clinicians or family)
i/mech	It sounds technical, mechanical, clinical (or not)	Emergent	More pleasant to me (given my personal taste)
i/pleasant	It is pleasant, likeable (or not) vs annoying irritating (or not)	ANSI/AAMI; 2018 [2]	

Table S4: How and why alarms were changed

Alarm	Example Excerpts	Parameter	Change	Benefit of Change	Risk of Change
Low Priority	• *Maybe more present but less frequent*<br>• *I would have it softer and less frequent*<br>• *[I wish] a slower pace of the beat could achieve the same level of attention/alarm*<br>• *I'm fine with the fact that this alarm is a little difficult to notice*<br>• *I wish there was more pause between beeps*<br>• *I wonder how it could be enriched to become less dull, less square, less strict*<br>• *[I wish] it would sound a bit more smooth and organic. Not so mechanic*<br>• *I wish they would be less technical, more human or more natural, like music*<br>• *reestablish a connection that 'this is a person we care about'*<br>• *I like it's pitch, it's in a good & useful range*<br>• *[I like that it's] distinguishable from other higher priority alerts.*	Interval	Increased	Reduced fatigue, more distinguishable from yellow	Potential to not hear (mitigated by note length increase)
		Amplitude envelope	Attack, sustain, and release increased. Overall length increased	Rounder, less machine-like, and less mistakable for a QRS tone	Potential to be mistaken for yellow (mitigated by yellow pitch increase)
		Pitch	Unchanged	Familiar, immediately recognizable	None
		Timbre	Unchanged	Familiar, immediately recognizable	None
Medium Priority	• *Could the interval be longer?*<br>• *It is the most common and constant alarm.... when mixing several monitors with the same sound is annoying*<br>• *if it's moderate priority I would want something to cue me in about the urgency*<br>• *We all tend to tune out what occurs repetitively*	Interval	Increased	Reduced fatigue, more distinguishable from cyan and red	Possibly mistaken as a lower priority (mitigated by even greater interval for cyan)
		Amplitude envelope	Sustain decreased, but overall length maintained.	More urgent (percussive) feel than cyan, with smoother tail	Potential not to hear (mitigated by pitch increase)

	• *My opinion is: change the INOP to the last "beep" sound (or maybe the yellow) and leave the red as is. the inop and yellow are too close in nature*<br>• *If I had a wish, Philips monitors should have a kind of harmony in their tones... that is a major not a minor harmony*<br>• *The INOP and yellow are too close in nature*<br>• *It gets your attention but not in an abrasive way*<br>• *Ideal world - still a sound that gains attention... but less harsh*	Pitch	Increased	More distinguishable from cyan	Potential for annoyance (mitigated by sustain decrease)
		Timbre	Harmonics removed	Less aggravating	Potential not to hear (mitigated by pitch increase)
**High Priority**	• *The red alarm is annoying, but it's supposed to be annoying*<br>• *On my ears this sound is a little annoying, but I know this is a red alarm & only plays for life threatening event & I need to look at my patient*<br>• *I'm hyper sensitive to it, which is good, but I wish there was some way where it wouldn't make me keep hearing it after the shift is over*<br>• *I can't stand the tone of it either.. the beeping & it's so loud creates anxiety just from hearing it, by the way it resonates*<br>• *It's like a bell ringing inside my head*<br>• *I'm very sensitive for the high frequencies*<br>• *Laboring patients who have preeclampsia can be affected and are potentially hypersensitive to seizures with this sound.*<br>• *I wish those alarms were more pleasant*	Interval	Unchanged (even though most interviewees stated that an increase would be OK)	Maintain feeling of urgency	None
		Amplitude envelope	Mostly the same	Keep highest priority alarm most recognizable	None
		Pitch	Unchanged	Familiar, immediately recognizable	None
		Timbre	Harmonic attenuated	Less aggravating	May be slightly less audible, but difference is negligible

Reference Multimedia Supplement 2

[1] Bloomenthal, A. What the Production Possibility Frontier (PPF) Curve Shows. *Investopedia*, 7 November 2021.

[2] Association for the Advancement of Medical Instrumentation. ANSI/AAMI HE75:2009 (R2018) Human Factors Engineering—Design of Medical Devices; American National Standards Institute Inc.: 2018.

[3] International Electrotechnical Commission. IEC 60601-1-8:2006+AMD1:2012+AMD2:2020 Medical Electrical Equipment; International Standards Organization: 2020.

[4] Bennett, C.; Dudaryk, R.; Crenshaw, N.; Edworthy, J.; McNeer, R. Recommendation of New Medical Alarms Based on Audibility, Identifiability, and Detectability in a Randomized, Simulation-Based Study. Crit. Care Med. 2019, 47, 1050–1057.

[5] Hasanain, B.; Boyd, A.D.; Edworthy, J.; Bolton, M.L. A formal approach to discovering simultaneous additive masking between auditory medical alarms. Appl. Ergon. 2017, 58, 500–514.

[6] Bolton, M.L.; Zheng, X.; Li, M.; Edworthy, J.R.; Boyd, A.D. An Experimental Validation of Masking in IEC 60601-1-8:2006-Compliant Alarm Sounds. Hum. Factors 2019, 001872081986291.

[7] Phansalkar, S.; Edworthy, J.; Hellier, E.; Seger, D.L.; Schedlbauer, A.; Avery, A.J.; Bates, D.W. A review of human factors principles for the design and implementation of medication safety alerts in clinical information systems. J. Am. Med. Inform. Assoc. 2010, 17, 493–501.

[8] Gillard, J.; Schutz, M. Composing alarms: Considering the musical aspects of auditory alarm design. Neurocase 2016, 22, 566–576.

[9] McDougall, S.; Edworthy, J. Soundscaping: Sound, meaning and vision in healthcare alarm systems. Proc. 32nd Int. BCS HCI Conf. 2018.

[10] McDougall, S.; Edworthy, J.; Sinimeri, D.; Goodliffe, J.; Bradley, D.; Foster, J. Searching for meaning in sound: Learning and interpreting alarm signals in visual environments. J. Exp. Psychol. Appl. 2020, 26, 89–107.

[11] Edworthy, J.R.; McNeer, R.R.; Bennett, C.L.; Dudaryk, R.; McDougall, S.J.P.; Schlesinger, J.J.; Bolton, M.L.; Edworthy, J.D.R.; Özcan, E.; Boyd, A.D.; Reid, S.K.J.; Rayo, M.F.; Wright, M.C.; Osborn, D. Getting Better Hospital Alarm Sounds Into a Global Standard. Ergon. Des. 2018, 26, 4–13.

[12] McNeer, R.R.; Horn, D.B.; Bennett, C.L.; Edworthy, J.R.; Dudaryk, R. Auditory Icon Alarms Are More Accurately and Quickly Identified than Current Standard Melodic Alarms in a Simulated Clinical Setting. Anesthesiology 2018, 129, 58–66.

[13] Bennett, C.; Dudaryk, R.; Crenshaw, N.; Edworthy, J.; McNeer, R. Recommendation of New Medical Alarms Based on Audibility, Identifiability, and Detectability in a Randomized, Simulation-Based Study. Crit. Care Med. 2019, 47, 1050–1057.

[14] Topf, M.; Dillon, E. Noise-induced stress as a predictor of burnout in critical care nurses. Heart Lung 1988, 17, 567–574.

[15] Topf, M. Hospital noise pollution: An environmental stress model to guide research and clinical interventions. J. Adv. Nurs. 2000, 31, 520–528.

[16] Morrison, W.E.; Haas, E.C.; Shaffner, D.H.; Garrett, E.S.; Fackler, J.C. Noise, stress, and annoyance in a pediatric intensive care unit. Crit. Care Med. 2003, 31, 113–119.

[17] Ryherd, E.E.; Waye, K.P.; Ljungkvist, L. Characterizing noise and perceived work environment in a neurological intensive care unit. J. Acoust. Soc. Am. 2008, 123, 747–756.

[18] Keller, J.P.; Diefes, R.; Graham, K.; Meyers, M.; Pelczarski, K. Why Clinical Alarms Are a 'Top Ten' Hazard: How You Can Help Reduce the Risk. Biomed. Instrum. Technol. 2011, 45, 17–23.

[19] Watson, J.; Kinstler, A.; Vidonish, W.P.; Wagner, M.; Lin, L.; Davis, K.G.; Kotowski, S.E.; Daraiseh, N.M. Impact of Noise on Nurses in Pediatric Intensive Care Units. Am. J. Crit. Care 2015, 24, 377–384.

[20] Cho, O.M.; Kim, H.; Lee, Y.W.; Cho, I. Clinical Alarms in Intensive Care Units: Perceived Obstacles of Alarm Management and Alarm Fatigue in Nurses. Healthc. Inform. Res. 2016, 22, 46–53.

[21] Basner, M. Noise: The Other Negative Effect of False Alarms. AAMI Alarms Summit, Herndon, VA, 2011.

[22] Shivers, J.P.; Mackowiak, L.; Anhalt, H.; Zisser, H. "Turn It Off!": Diabetes Device Alarm Fatigue Considerations for the Present and the Future. J. Diabetes Sci. Technol. 2013, 7, 789–794.

[23] Basner, M.; Babisch, W.; Davis, A.; Brink, M.; Clark, C.; Janssen, S.; Stansfeld, S. Auditory and non-auditory effects of noise on health. Lancet 2014, 383, 1325–1332.

[24] Sakallaris, B.R.; MacAllister, L.; Voss, M.; Smith, K.; Jonas, W.B. Optimal Healing Environments. Glob. Adv. Health Med. 2015, 4, 40–45.

[25] Sen, Y.; Sen, A. The Future of Hospital Sound: Transforming Healthcare Through Sound Experience. Music Med. 2020, 12, 5.

[26] Murthy, V.S.; Malhotra, S.K.; Bala, I.; Raghunathan, M. Detrimental effects of noise on anaesthetists. Can. J. Anaesth. 1995, 42, 608–611.

[27] Hasfeldt, D.; Laerkner, E.; Birkelund, R. Noise in the Operating Room—What Do We Know? A Review of the Literature. J. Perianesth. Nurs. 2010, 25, 380–386.

[28] Keller, S.; Tschan, F.; Beldi, G.; Kurmann, A.; Candinas, D.; Semmer, N.K. Noise peaks influence communication in the operating room: An observational study. Ergonomics 2016, 59, 1541–1552.
